# Palpebral leishmaniasis

**DOI:** 10.1002/ccr3.9197

**Published:** 2024-07-16

**Authors:** Mohammad Sharifi, Tayebe Shiravi, Ali Bolouki, Mehrdad Motamed Shariati

**Affiliations:** ^1^ Eye Research Center Mashhad University of Medical Sciences Mashhad Iran

**Keywords:** direct smear, leishmaniasis, palpebral ulcer, scar

## Abstract

Leishmaniasis can mimic many conditions, including hordeolum, basal cell carcinoma, and squamous cell carcinoma. The presence of kinetoplast in free‐form or intramacrophage amastigotes, ensuring us to establish the microscopic diagnosis of leishmaniasis.

## 1 CASE PRESENTATION

**FIGURE 1:**
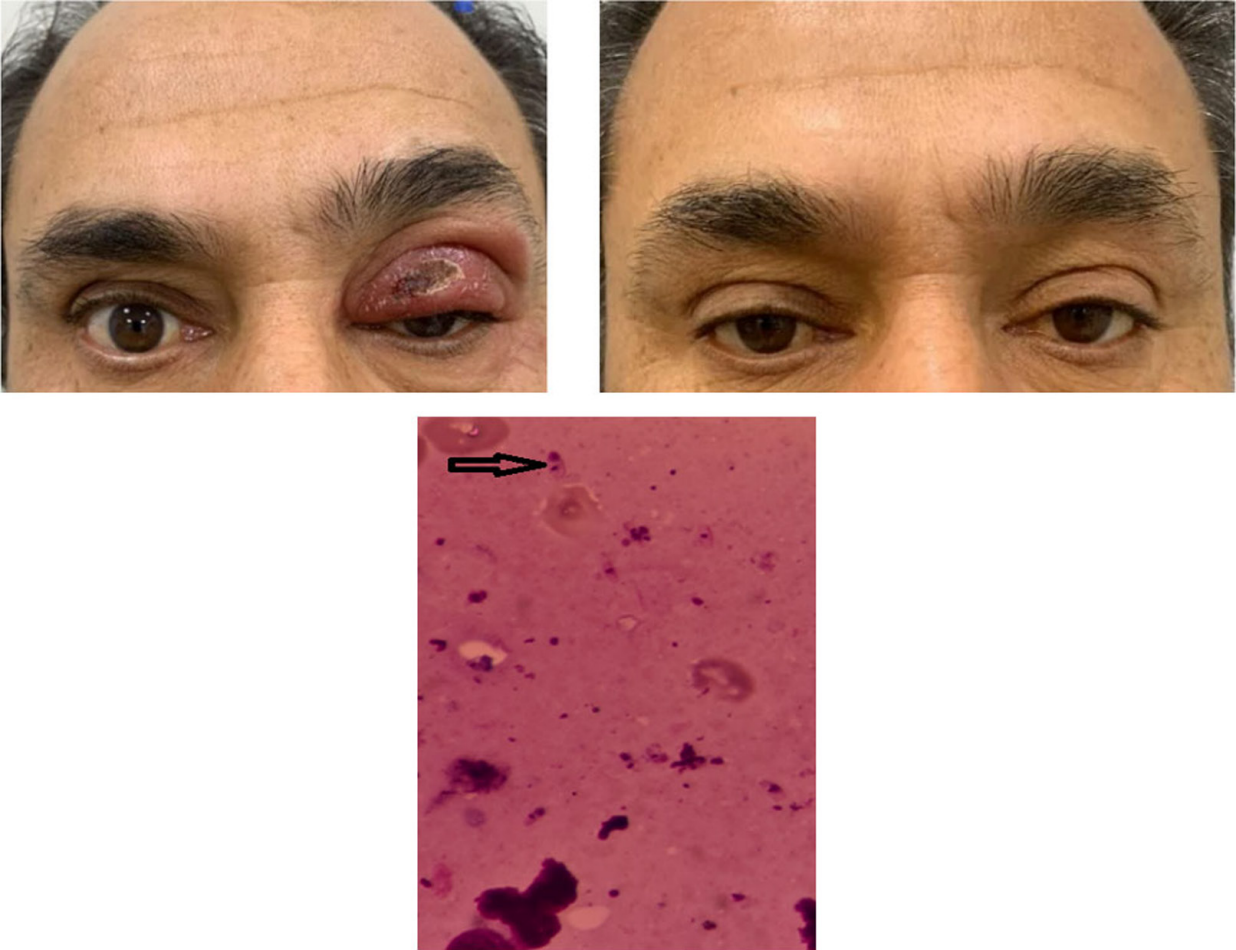
Ocular leishmanial lesion involving the left upper eyelid before the treatment (left upper) and 6 months after treatment (right upper). Direct smear from the skin ulcer shows amastigotes in the cytoplasm of macrophages (lower image, black arrow).

A 50 y/o man presented with a progressive left upper eyelid skin ulcer since 6 months ago. During this period, he received topical and systemic antibiotics with the diagnosis of skin bacterial infection. On the examination, a 10 × 15 mm lesion was observed, which involved the left upper eyelid (Figure 1). Slit lamp examination, fundus examination, and intraocular pressures were unremarkable. Visual acuity was 10/10 in the eyes. Systemic examination excluded lymphadenopathy.

Considering the endemicity of leishmania in the area where the patient lived, Mashhad, we suspected leishmaniasis. A direct smear of the lesion revealed Leishman bodies. Pathology examination showed leishmania organisms within and next to macrophages (Figure 1). Also, we suggested the PCR test. However, the patient refused to do it. The patient was diagnosed with palpebral leishmaniosis and treated with systemic Glucantime (20 mg/kg) daily for 3 weeks. The option of treatment with intralesional injections of meglumine antimoniate was offered to the patient. He refused local treatment.

## 2 DISCUSSION

Leishmaniasis is classified into Old and New World cutaneous leishmaniasis, mucocutaneous and visceral leishmaniasis. Cutaneous leishmaniasis is characterized by single or multiple ulcerative skin lesions, usually on the face or extremities. Cutaneous leishmaniasis is common in Iran and is usually caused by *L*. *major* or *L*. *tropica*. Leishmania is transmitted by a sandfly infected with leishmania parasites. Infection of the eyelid is rare because eyelashes and eyelid movements usually prevent the sandfly from biting the skin of there. Adnexal leishmaniosis is a challenging diagnosis in non‐endemic areas and is not accompanied by any cutaneous lesions elsewhere on the body.^1^

Leishmaniasis can mimic many conditions, including chronic blepharitis, hordeolum, basal cell carcinoma, and squamous cell carcinoma, so we need a high index of suspicion to diagnose it immediately. Palpebral involvement can lead to ectropion and trichiasis, emphasizing early diagnosis and treatment Cutaneous leishmaniasis is usually a self‐limiting disease. However, if left untreated, eyelid leishmaniasis can spread to the conjunctiva and cornea, causing conjunctivitis and interstitial keratitis.^2^

Treatment of ocular leishmaniasis includes systemic meglumine antimoniate 20 mg/kg for at least 3 weeks and allopurinol. Intralesional meglumine antimoniate can also be used twice weekly for at least 2 weeks. Another choice is paromomycin ointment. Also, intra‐lesion injection of meglumine antimoniate is effective as a systemic treatment but is more effective than paromomycin ointment.^3^

One of the important points in the mentioned case is the absence of scars after recovery. A scar on the edge of the eyelid can lead to complications such as ectropion and exposure keratopathy.

## AUTHOR CONTRIBUTIONS

**Mohammad Sharifi:** Investigation; writing – review and editing. **Tayebe Shiravi:** Data curation; writing – original draft. **Ali Bolouki:** Data curation; writing – original draft; writing – review and editing. **Mehrdad Motamed Shariati:** Conceptualization; data curation; supervision; writing – original draft; writing – review and editing.

## FUNDING INFORMATION

The authors received no funding.

## CONFLICT OF INTEREST STATEMENT

The authors declare that they have no competing interests.

## CONSENT

Written informed consent was obtained from the patient to publish this report in accordance with the journal's patient consent policy.

## Data Availability

The data that support the findings of this study are available on request from the corresponding author. The data are not publicly available due to privacy or ethical restrictions.
